# Assessing Human Judgment of Computationally Generated Swarming Behavior

**DOI:** 10.3389/frobt.2018.00013

**Published:** 2018-02-22

**Authors:** John Harvey, Kathryn Elizabeth Merrick, Hussein A. Abbass

**Affiliations:** ^1^School of Engineering and Information Technology, University of New South Wales, Canberra, ACT, Australia

**Keywords:** swarming, flocking, perception of biological motion, swarm intelligence, human perception

## Abstract

Computer-based swarm systems, aiming to replicate the flocking behavior of birds, were first introduced by Reynolds in 1987. In his initial work, Reynolds noted that while it was difficult to quantify the dynamics of the behavior from the model, observers of his model immediately recognized them as a representation of a natural flock. Considerable analysis has been conducted since then on quantifying the dynamics of flocking/swarming behavior. However, no systematic analysis has been conducted on human identification of swarming. In this paper, we assess subjects’ assessment of the behavior of a simplified version of Reynolds’ model. Factors that affect the identification of swarming are discussed and future applications of the resulting models are proposed. Differences in decision times for swarming-related questions asked during the study indicate that different brain mechanisms may be involved in different elements of the behavior assessment task. The relatively simple but finely tunable model used in this study provides a useful methodology for assessing individual human judgment of swarming behavior.

## 1 Introduction

This paper uses a relatively simple computer-based model to, as Shmueli et al. (2014) put it, help us “sense, understand, and shape human behavior.” Following the broad approach of Servi and Elson (2014), the aim is to provide “… a mathematically unbiased approach … ” for understanding human judgment of swarming behavior.

Computer-based swarm systems, aiming to replicate the flocking behavior of birds, were first introduced by Reynolds (1987). Reynolds’ “boids”, short for “birdoids,” were based on the emergent behavior resulting from the interaction of three simple rules: attraction, alignment and repulsion. Reynolds (1987) noted that while the dynamics of the resulting behavior was difficult to quantify, people who viewed them “… immediately recognized them as a representation of a natural flock.” Understanding individual behavior, and the differences between the behavior of individuals, will contribute to understanding and potentially shaping human behavior overall.

Toner and Tu (1998) define a flock as “the collective coherent motion of large numbers of self-propelled organisms.” “Flocking” is one of many terms used to describe a form of behavior referred to as “collective motion”. Vicsek and Zafeiris (2012) define collective motion as “… a phenomenon occurring in collections of similar, interacting units moving with about the same absolute velocity.” Collective motion includes the behaviors known as flocking, swarming, schooling, shoaling, and herding. The term “flocking” generally applies to birds, “shoaling” and “schooling” to fish, and “herding” to land animals.

The term “swarming” is applied in a broad sense, being applied to collections of like elements more broadly than just animals—including people, unmanned aerial vehicles, robots, etc.—and will be used for the current study. Clough (2002) defined a swarm as a “ … collection of autonomous individuals relying on local sensing and reactive behaviors interacting such that a global behavior emerges from the interactions.” As Clough noted, a common feature of systems displaying swarming behavior is that the dynamics is an emergent phenomenon, with structure at the macro level resulting from interactions at the micro level.

The key features of swarming behavior are as follows:

- there are large numbers of like particles which are clustered or grouped, and
- particles are continuously moving, but their motion is not uniformly ordered or aligned, i.e., the individual particles move in diverse directions.

Overall, the effect is one of dynamic stability with a balance between regular and random shape and motion that human observers recognize as “natural” or “life-like”.

There has been extensive quantitative study of many examples of swarming behavior, and a good summary of the field is provided in the study by Vicsek and Zafeiris (2012). Wu et al. (2011) have suggested that the emergent properties of swarming systems occur when the systems are “at the edge of chaos.” Das et al. (2012) have demonstrated chaotic system dynamics in a social foraging swarm model, and the range of parameters for which chaos exists in the dynamics was quantified. They also showed that swarm parameters can be chosen such that the swarm can be stable, convergent, or chaotic. Similarities with chaotic systems include the emergence of structure without explicit external control, the existence of multistability (coexistence of many stable states), and the existence of state transitions with dramatic change in the system dynamics. Mecholsky et al. (2012) investigated a broad range of control parameters to determine the stability of models of flocks. In related work, Harvey et al. (2015) applied quantitative measures used to characterize chaotic systems to a simplified version of Reynolds’ boids model to identify the range of parameter values for which swarming would occur. There has not, however, been any systematic analysis of the identification of swarming by human observers. Nor has there been any systematic attempt to develop a mathematical model of subjective identification of swarming behavior.

The aim of this paper is to extend the work in Harvey et al. (2015) to determine the conditions under which swarming is identified by human observers using a computationally generated swarm model. The motivation of this work is twofold. First, if the quantitative metrics of the identification of swarming could be developed to perfectly describe humans’ perception of swarming, one could use these metrics during an evolutionary process to discover new rules and models for swarming without expensive and tedious human evaluations. This could be useful in automatic generation of visual effects. Second, swarming has been a subjective topic without proper mechanisms to explain its root causes or a proper quantification of the phenomenon. The ability to understand the root causes of humans’ identification of swarming contribute to our objective understanding of the phenomenon.

Decision times to make “swarming” and related “group” and “order” decisions are analyzed to help understand the basis of the decisions involved. The key advantage of the computationally generated swarm model used in this study is that dynamics can be directly and finely controlled using the model control parameters.

This article is organized as follows. In Section 2, the experimental method is explained. Results are presented and discussed in Section 3. Conclusions are drawn in Section 4 along with a discussion on future work.

## 2 Experimental Method

The aim of the subjective study was to identify the range of control parameters for a simplified boids model for which swarming behavior is identified by human subjects. Based on the work in Harvey et al. (2015), it was hypothesized that subjects would identify swarming behavior in the test cases classified as “swarming cases” using objective measures. Conversely, subjects would identify behavior as “not swarming” for the cases classified as “non-swarming cases” using the same objective measures. No significant difference in performance between subjects was expected. It was also hypothesized that when subjects identified behavior as swarming they would also identify that the particles were “grouped” and their motion was “not ordered”, consistent with the results of objective studies.

In the remainder of this section, the simplified boids model used for the subjective study is defined (Section 2.1) and the experimental method described in detail (Section 2.2). Results are then presented and discussed (Sections 3.1 and 3.2).

### 2.1 Boids Model

The boids model used for the current study is based on the original work by Reynolds (1987) as modified by Harvey et al. (2015). The model uses three simple rules that lead to simulated swarming behavior, which Reynolds lists in decreasing precedence as:

- Collision avoidance (repel): avoid collision with nearby flockmates.
- Velocity matching (align): attempt to match velocity with nearby flockmates.
- Flock centering (attract): attempt to stay close to nearby flockmates.

The initial location and velocity of each particle are selected at random from a uniform distribution. All other aspects of the boids model are fully deterministic and repeatable. A key point, not explicit in the original Reynolds paper, is that the rules and associated parameters applying to the particles are the same for all particles, i.e., the population is homogenous.

To simplify the model, neither aerodynamics nor gravity is simulated. Particles are defined as points in space, and it is therefore possible for particles to overlap or for one particle to “pass through” another if there is no repulsion force in effect. There are no directional constraints on senses, particles are therefore able to sense distance from other particles in all directions. Attraction, repulsion, and alignment forces apply to “nearby” particles, where nearby is defined using a control parameter that specifies a range. Particles inside the range are considered nearby while those outside the range are not. Particles are “reflected” when they reach the boundaries of the experimental area. Neither obstacle avoidance nor a goal seeking force is considered. Motion is only considered in two dimensions.

A detailed mathematical description of the implementation of the simplified boids model is in the study by Harvey et al. (2015). The current study builds on the original study in Harvey et al. (2015) by adding subjective assessment of particle behavior and using an expanded range of control parameters for the simplified boids model, as shown in Table 1. These parameters were identified in the previous study to produce stable swarming behaviors within the allocated number of iterations.

**Table 1:** Experiment control parameters.

Parameter	Description	Value
*N*	Total number of particles	100
*R_max_*	Region size	500
*V_max_*	Maximum velocity	1, 2, 4
*T*	Total iterations	1500
*R_c_*	Attraction range	100
*R_a_*	Alignment range	100
*R_s_*	Repulsion range	10
1/*F_FT_*	Flock together factor	0, 0.005, 0.01, 0.02
1/*F_RV_*	Relative velocity factor	0, 0.0625, 0.125
1/*F_KA_*	Keep away factor	0, 0.50, 1, 2

*Values used in the original study by Harvey et al. (2015) are underlined*.

**Figure 1:**
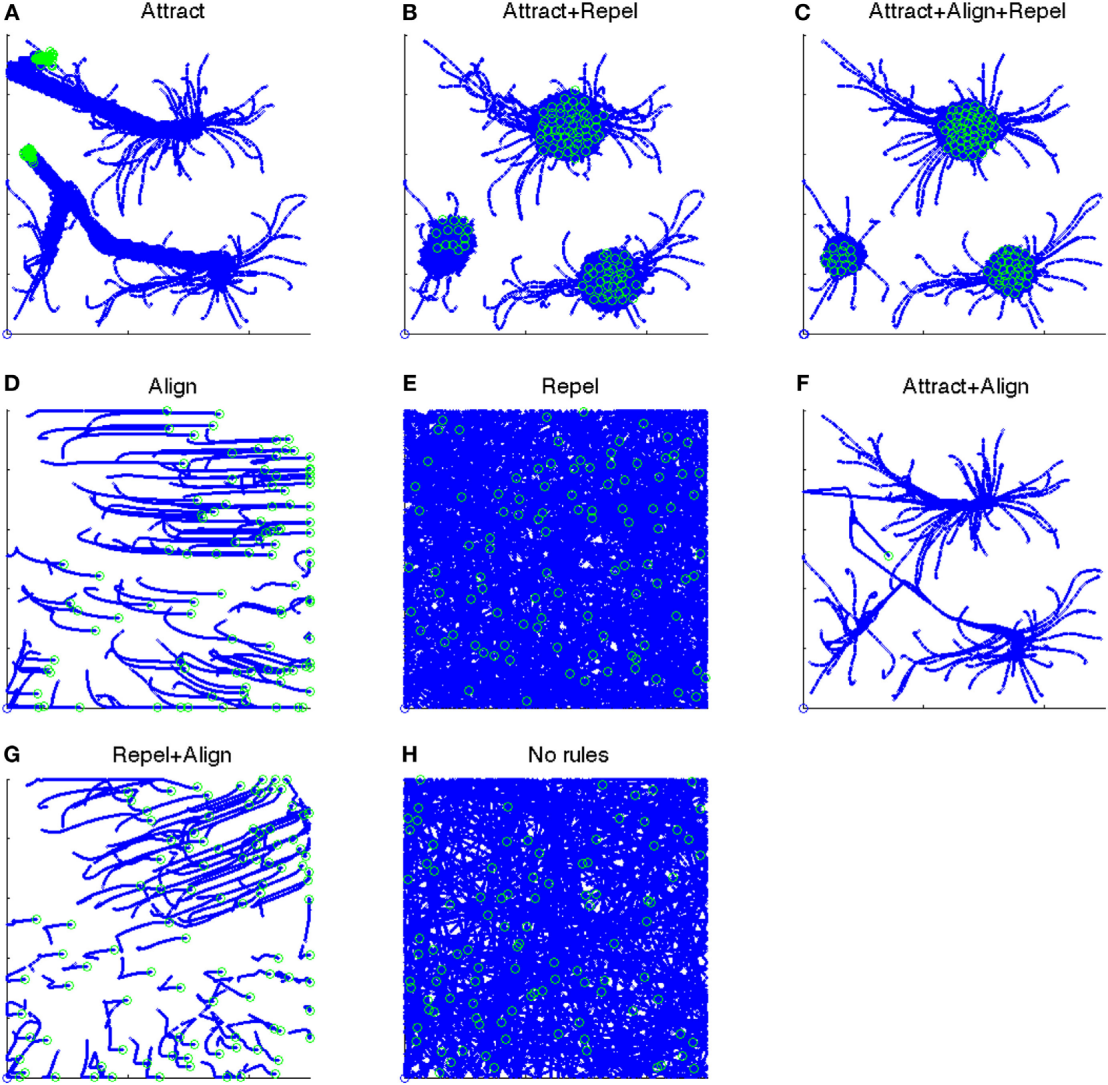
Particle paths for eight test cases from *t* = 501:1,500. Position at *t* = 501 shown as an open blue circle. Position at *t* = 1,500 shown as an open green circle. **(A)** The particle paths for the “Attract” case, i.e., when only the Attract force is present. **(B)** The particle paths for the “Attract + Repel” case, i.e., when both the Attract and Repel forces are present. **(C)** The particle paths for the “Attract + Repel + Align” case, i.e., when the Attract, Repel, and Align forces are present. **(D)** The particle paths for the “Align” case, i.e., when only the Align force is present. **(E)** The particle paths for the “Repel” case, i.e., when only the Repel force is present. **(F)** The particle paths for the “Attract + Align” case, i.e., when both the Attract and Align forces are present. **(G)** The particle paths for the “Repel + Align” case, i.e., when both the Repel and Align forces are present. **(H)** The particle paths for the “No rules” case, i.e., when no forces are present.

The 1/*F_FT_*, 1/*F_RV_*, and 1/*F_KA_* control parameters determine whether the attract, align, and repel forces are applied and their strength. The presence or absence of these three forces results in eight “rule-sets”, as shown in Table 2. A particle path diagram for the 100 boids for one example of each rule-set for *V_max_* = 2.0 is shown in Figure 1.

**Table 2:** Definition of rule-sets—based on presence or absence of control parameters for attract, align, and repel forces.

1/*F_FT_*(attract)	1/*F_RV_*(align)	1/*F_KA_*(repel)	Rule-set
>0	0	0	Attract
>0	0	>0	Attract + repel
>0	>0	>0	Attract + repel + align
>0	>0	0	Attract + align
0	>0	0	Align
0	0	>0	Repel
0	>0	>0	Align + repel
0	0	0	No rules

The rule-sets determine whether or not particles are “grouped” and “ordered”, and hence whether behavior is swarming or not-swarming, as shown in Table 3. Harvey et al. (2015) found that three rule-sets—Attract, Attract + Repel, and Attract + Align + Repel—result in swarming behavior, based on objective measures. No other rule-sets displayed swarming behavior.

**Table 3:** Behavioral characteristics of rule-sets, categorized by “grouping” and “order”.

	Grouped	Not grouped
Not ordered	**Attract**	Repel
	**Attract + Repel**	No rules
	**Attract + Align + Repel**	

Ordered	Attract + Align	Align
		Align + Repel

*Objectively identified “swarming cases” are in bold*.

### 2.2 Experimental Protocol

**Figure 2:**
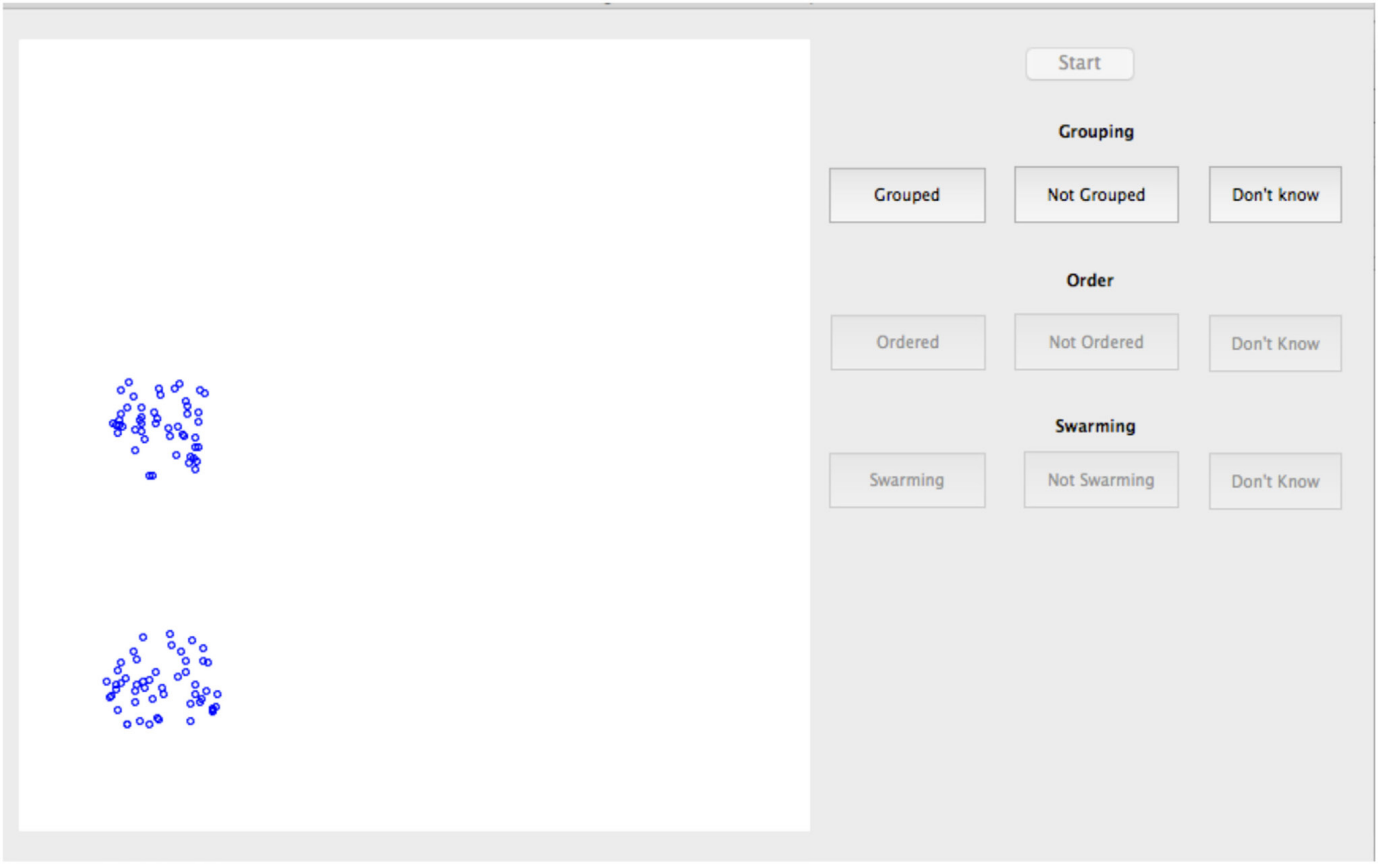
Screenshot of subjective swarming test.

The study used a laptop computer with a presentation area for the model of 11 cm × 11 cm viewed from a distance of approximately 60 cm. The display layout is as shown in Figure 2. Individual particles are represented by a 5 pixel diameter blue circle (approximately 0.11 mm on the screen) on a white background. Explicit heading orientation of the particles is not shown. Particles were displayed for iterations from *t* = 501:1,500. Presentation rate was held constant for all cases at approximately 20 iterations (position updates) per second. As shown in Table 1, the range of control parameters comprised three values each of *V_max_* and 1/*F_RV_* and four values each for 1/*F_FT_* and 1/*F_KA_*. This provided a total of 144 test cases for examination.

#### 2.2.1 Participants

There were 30 participants in the study, 17 males and 13 females. Subject ages ranged from 21 to 82 years. All participants gave informed consent, and the UNSW Canberra Human Research Ethics Advisory Panel approved the experiment protocol.

#### 2.2.2 Design

The structure of the study is shown in Table 4. The 144 test cases were arranged in a random order (but the same for all subjects) and presented to subjects in two groups of 72 each. Three calibration sessions—where subjects were directed to make a specific response—were included in the study. The aim of the calibration sessions was to allow calculation of the subjects’ simple response time without having to make a decision.

**Table 4:** Test case presentation.

Presentation	Description
1:5	Calibration Set 1
6:77	First 72 test cases
78:82	Calibration Set 2
83 – 154	Second 72 test cases
155 – 159	Calibration Set 3

For each of the test cases, subjects were asked three questions about the dynamics of the particles in each presentation as follows:

- **Swarming question**: *does the motion of the particles appear to be life-like, swarming motion, like a group of birds or bees or flies or insects?* Possible responses to this question were “swarming”, “not swarming”, and “don’t know”.
- **Grouping question**: *are the particles grouped, i.e., clustered together, or are they scattered across the experiment area?* Possible responses were “grouped”, “not grouped”, and “don’t know”.
- **Order question**: *are the particles ordered, i.e., are they generally all moving in the same direction or are they moving in different directions?* Possible responses were “ordered”, “not ordered”, and “don’t know”.

The sequence of the questions was varied such that each of the three questions was asked first for one third of the subjects to balance any order effect on decision time. Particle motion was not stopped until the third question was answered. Subjects were then required to select a “start” button to start the next presentation. The total study took between 25 and 40 min for each subject.

#### 2.2.3 Data Recorded

Subject data recorded comprised the age and sex of subject. Response data recorded from each subject comprised the response to each of the “group”, “order”, and “swarm” questions as well as the time taken to make each response. All other data were derived from this raw data.

## 3 Results and Discussion

### 3.1 Judgment of Swarming Behavior

#### 3.1.1 Average Results across All Subjects

**Figure 3:**
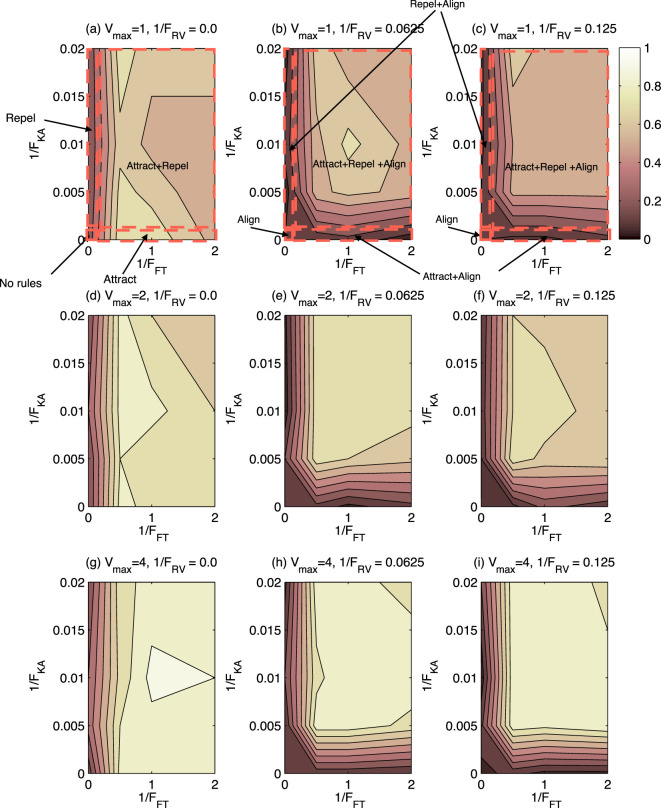
Contour plot of proportion of subjects choosing “swarming” for 144 test cases. Lighter shading indicates higher proportion of subjects identifying swarming behavior. Rule-sets are shown for *V*_max_ = 1.0. **(A)**–**(C)** The cases when *V*_max_ = 1 and 1/F_RV_ = 0.0, 0.0625, and 0.125, respectively. **(D)**–**(F)** The cases when *V*_max_ = 2 and 1/F_RV_ = 0.0, 0.0625, and 0.125, respectively. **(G)**–**(I)** The cases when *V*_max_ = 4 and 1/F_RV_ = 0.0, 0.0625, and 0.125, respectively.

Figure 3 shows the proportion of subjects identifying behavior as “swarming” for the 144 test cases presented. As shown in Figure 3, there is a wide range of test cases for which swarming behavior is identified. In no case, however, do 100% of subjects identify that swarming is occurring, the maximum case being approximately 90%. Nor is there a case where the proportion of subjects identifying swarming is 0%, the minimum being approximately 5%. These findings show that a decision on swarming or not-swarming is not a clear cut decision, but varies considerably between test cases and individuals.

**Figure 4:**
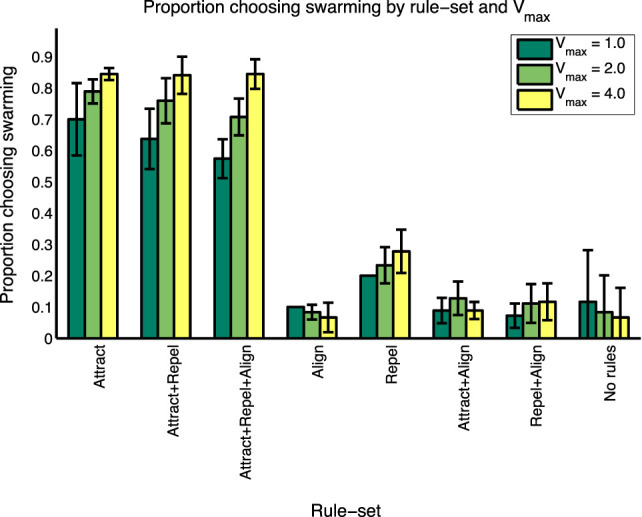
Summary results of proportion choosing “swarming” by rule-set and *V_max_*.

To better understand the conditions under which swarming was identified, the 144 test cases were categorized into the eight “rule-sets” used in the study by Harvey et al. (2015) and as shown in Table 2. The results of this analysis are shown in Table 5 and Figure 4.

**Table 5:** Total test cases classified by rule-set and corresponding proportion of subjects choosing “swarming.”

	Rule-set	Total	Proportion choosing swarming
Swarmingcases	Attract only	9	0.78
	Attract + Repel	27	0.75
	Attract + Align + Repel	54	0.71
Non-swarming cases	Align only	6	0.08
	Repel only	9	0.24
	Attract + Align	18	0.10
	Repel + Align	18	0.10
	No rules	3	0.09
Summary	Swarming cases	90	0.73
	Non-swarming cases	54	0.13

As shown in Table 5, and consistent with the findings in the study by Harvey et al. (2015), not all of Reynolds’ three forces are necessary for swarming behavior to occur. A majority of subjects identify that swarming is occurring in all instances of three rule-sets: Attract, Attract + Repel, and Attract + Repel + Align. These three rule-sets—the “swarming cases”—comprise 90 of the 144 test cases. The average proportion of subjects identifying swarming behavior for each of these rule-sets is significantly higher (ANOVA, *p* < 0.001) than for each of the other five rule-sets, the “non-swarming cases”.

To further investigate the range of responses, Figure 2 shows the swarming responses classified by rule-set and the three values of *V_max_*. Balanced one-way analysis of variance [ANOVA, see Fisher (1925) for an explanation] of the three swarming cases shows that, for the swarming cases, the proportion of subjects identifying swarming behavior is significantly higher for *V_max_* = 4.0 compared with *V_max_* = 1.0 (*p* < 0.05). *V_max_* does not have a significant effect on subject responses for the non-swarming cases.

The breakdown by rule-set and *V_max_* shows that subjects’ identification of swarming behavior is sensitive to both the rule-sets and the maximum velocity that control the behavior of the particles. However, it is necessary to look at individual responses to better understand the distribution of results.

#### 3.1.2 Individual Responses

**Figure 5:**
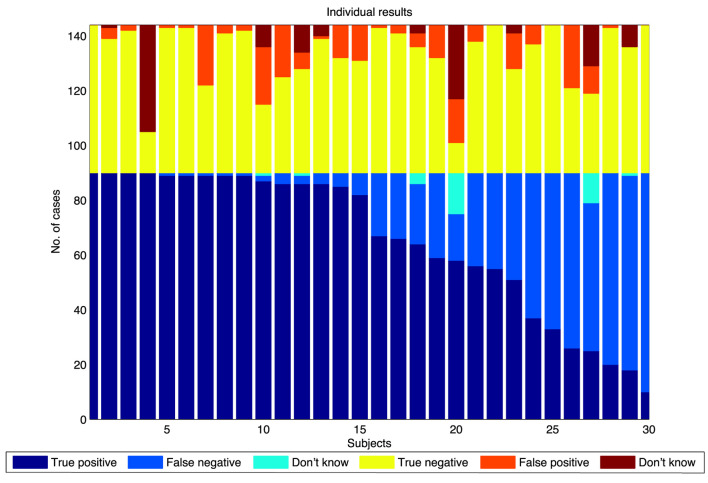
Swarm decision results for individuals, sorted by number of “true positive” responses.

Figure 5 shows the “swarming” results for individual subjects, grouped into swarming cases and non-swarming cases. Responses are categorized as true positives (identifying “swarming” for the swarming cases), false negatives (identifying “swarming” for the non-swarming cases), and “don’t know” for the swarming cases. Responses are classified as true negative (identifying “not swarming” for the non-swarming cases), false positive (identifying “swarming” for the non-swarming cases), and “don’t know” for the non-swarming cases.

As shown in Figure 5, there is a wide range of individual responses in identifying swarming behavior rather than an average level of response across all subjects. These results lead to two hypotheses:

1. there are different degrees of “swarminess” within the swarming cases for this model and
2. individual subjects have different “swarminess” thresholds for identifying behavior as swarming.

#### 3.1.3 Effect of *V_max_* on Individual Responses

**Figure 6:**
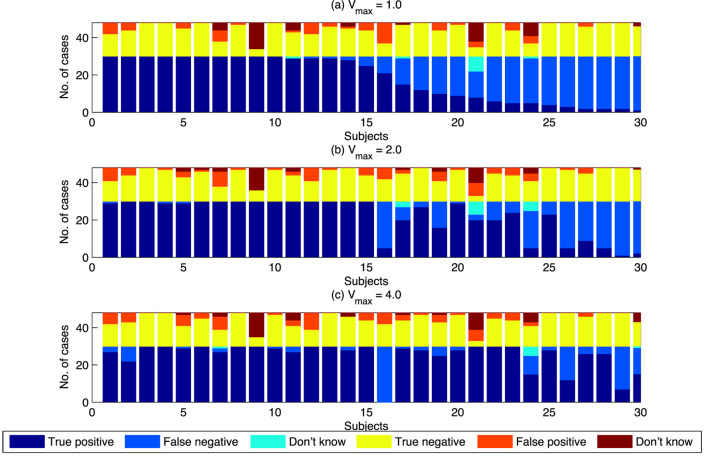
Swarm decision results for individuals, classified by *V*_max_. Subjects are sorted by the proportion of “true positives” for the *V*_max_ = 1.0 case. **(A)** The case where *V*_max_ = 1. **(B)** The case where *V*_max_ = 2. **(C)** The case where *V*_max_ = 4.

Results for individuals were categorized into the three values of *V_max_*. The results are shown in Table 6 and Figure 6. As shown in Table 6, there is a significant increase in the proportion of true positives for the swarming cases as *V_max_* increases. Balanced one-way ANOVA shows that results for *V_max_* = 4.0 are significantly higher than for *V_max_* = 1.0 (*p* < 0.05).

**Table 6:** Individual response levels categorized by *V_max_*, shown as a percentage of total responses for swarming and non-swarming cases.

		*V_max_*(%)		
	Condition	1.0	2.0	4.0
Swarming	True positive	61	73	84
	False negative	38	25	15
	Don’t know	1	2	1

Not swarming	True negative	80	80	78
	False positive	12	14	14
	Don’t know	8	6	8

Figure 6 shows that the overall increase in true positives is not uniform across all individuals. A large number of individuals achieve 100% true positives for *V_max_* = 1.0. The increase in true positives as *V_max_* increases is the result of individuals with lower levels of true positives at *V_max_* = 1.0 achieving higher levels of true positives as *V_max_* increases. *V_max_*, therefore, is a determinant of “swarminess” and for some subjects’ decision to identify behavior as swarming. Increase in true positives as *V_max_* increases is not always the case. As shown in Figure 6, for Subject 16 the proportion of true positives decreases as *V_max_* increases. The swarm model used in this study provides a mechanism to further investigate individual differences that lead to differences in the identification of swarming.

#### 3.1.4 Effect of Sex of Subject on Individual Responses

**Figure 7:**
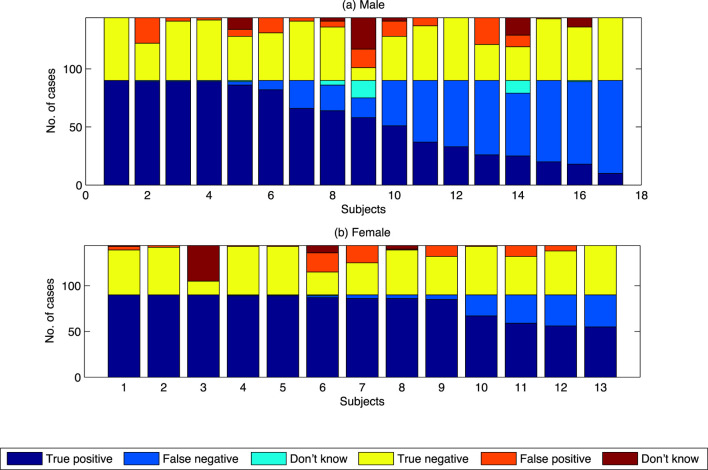
Summary results of proportion choosing “swarming” by class by sex of subject. **(A)** Results for Male subject. **(B)** Results for Female subjects.

The 30 subjects in the study were divided into two groups based on the sex of the subject. Results of the analysis are shown in Table 7 and Figure 7. Unbalanced one-way ANOVA of the results in Table 7 showed that female subjects displayed a higher proportion of true positives (and correlated lower number of false negatives) than the male subjects (*p* < 0.05). There was no difference in proportion of subjectives choosing swarming between males and females for the true negatives/false positives.

**Table 7:** Individual performance categorized by sex of subject.

	Condition	Male (%)	Female (%)
Swarming	True positive	60	88
	False negative	37	12
	Don’t know	2	0

Not swarming	True negative	79	81
	False positive	14	11
	Don’t know	7	7

Differences in proportion of subjects choosing swarming between males and females have been found in other studies of visual processing tasks. For example in the studies by Thayer and Johnsen (2000) and Montagne et al. (2005), females were found to be more accurate than men in recognizing facial expressions. Similar results were found in the study by Campbell et al. (2002), where women were more accurate than men in recognition of expressions of “disgust” and “anger.” Investigating the processing of facial expressions, Natale et al. (1983) also found there where differences in where the processing was done. These lateralization effects, i.e., the differences between males and females in their brain’s tendency to show more dominant cognitive processing in one hemisphere than the other, were also found by Campbell et al. (2002). In analyzing these and related results, Voyer (1996) found clear brain lateralization effects across a range of visual and verbal processing tasks. Voyer concluded that the differences between men and women could be explained, at least in part, by the fact that men tend to be more lateralized than women. The computer-based model used in this study provides the opportunity to further investigate sex-based and/or lateralization-based differences between individuals using a tunable, quantifiable model.

#### 3.1.5 Analysis by Sex of Subject and *V_max_*

Results were also assessed for the combined effect of sex of subject and *V_max_*. Results are shown in Table 8. Analysis of covariance of sex of subject and *V_max_* showed that the interaction is not significant, i.e., both male and female subjects’ proportion of true positives increase as *V_max_* increases.

**Table 8:** Average number of true positives by sex of subject and *V_max_*.

*V_max_*	All	Male	Female	Significant difference (*p* < 0.05)
1	0.61	0.50	0.74	No
2	0.73	0.58	0.93	Yes
4	0.84	0.74	0.97	Yes

Overall	0.73	0.61	0.88	Yes

#### 3.1.6 Effect of Age on Individual Responses

The total sample of 30 subjects was divided into three equal groups of 10 based on age. The summary results are shown in Table 9. Balanced one-way ANOVA of the results in Table 9 showed that there was no significant difference in response levels for the swarming cases based on age, other than the existence of a higher number of “don’t know” responses for the youngest age group for the non-swarming cases (*p* < 0.001). The results suggest that there is a higher willingness for younger subjects to make a “don’t know” response than for the older age groups.

**Table 9:** Individual performance by age.

		Age (%)		
	Condition	≤31 years	>31 ≤ 53 years	>53 years
Swarming	True positive	77	71	71
	False negative	20	29	29
	Don’t know	3	0	0

Not swarming	True negative	61	87	90
	False positive	18	12	9
	Don’t know	21	1	1

### 3.2 Judgment of “Group” and “Order”

**Figure 8:**
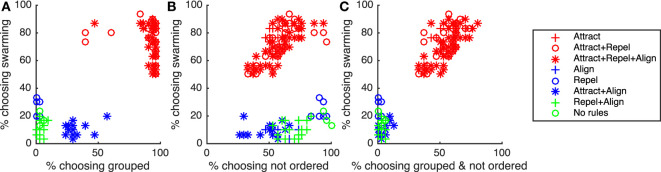
Correlation between proportion of subjects choosing “swarming” and **(A)** proportion of subjects choosing “grouped”, **(B)** proportion of subjects choosing “not ordered”, and **(C)** proportion of subjects choosing “grouped & not ordered”. “Swarming cases” are in red, “non-swarming” cases are in blue and green.

As noted in Section 1, for swarming to be present, particles need to be both grouped and exhibiting ongoing, unordered motion. Subjects identifying behavior as “swarming” were therefore expected to identify the behavior as both “grouped” and “not ordered.” Figures 8A–C and Table 10 show the results of correlation analysis of the relationship between the proportion of subjects choosing “swarming” and responses to the “group” and “order” questions and the combination of the two.

**Table 10:** *R*^2^-value of correlation between proportion of subjects choosing “swarming” and results of “grouped” and “ordered” questions.

Measures	All	Male	Female
“Grouped”	0.77*	0.64*	0.81*
“Not ordered”	0.00	0.00	0.00
“Grouped & not ordered”	0.91*	0.74*	0.86*

Correlations that have p < 0.05 are marked with a “*”.

As shown in Table 10, there is a strong positive correlation between subjects identifying behavior as “swarming” and “grouped”. While necessary for the existence of swarming behavior, being grouped is not sufficient. All instances of the non-swarming Attract + Align rule-set also result in particles being grouped, coalescing into 1, 2, or 3 overlapping elements. As shown in Figure 8A, generally 50% or fewer of subjects identified the Attract + Align rule-set as “grouped”. Subjects appear to treat these overlapping groups as a small number of individual particles rather than a small number of very tightly grouped particles. For future studies, if required, the use of a small repel force, and a small repel radius would provide sufficient separation of particles to allow identification of grouping.

For the “order” question, as shown in Table 10, there is no significant correlation between the proportion of subjects choosing “swarming” and proportion of subjects choosing “not ordered”. Examination of Figure 8B shows that unlike the “group” question there is a broad spread of responses to the “order” question and no clear differentiation between the swarming and non-swarming cases.

The combination of the two measures, as shown in Table 10 and Figure 8C, results in a very strong positive correlation and clear separation between the swarming and non-swarming cases. This is because the swarming and non-swarming test-cases that were overlapping in the “group” question are generally not overlapping in the “order” question and *vice versa*. The results from the study support the hypothesis that subjects that identify behavior as swarming also identify it as both “grouped” and “not ordered”. Decisions on “group” and “order” are therefore likely to be key contributing factors for subjects identifying whether behavior is swarming or not swarming.

### 3.3 Decision Times

Decision times for the “group”, “order,” and “swarm” questions were analyzed for each subject to determine whether there was any significant difference in subject decision times for each of the test cases. A decision time (*T_d_*) was calculated from the recorded response time (*T_r_*), as shown in Equation 1.

(1) $$T_{d}^{i}=T_{r}^{i}-T_{s}^{i}$$

where $T_{d}^{i}$ = decision time for the *i*th question, $T_{r}^{i}$ = response time for the *i*th question, and $T_{s}^{i}$ = simple response time for the *i*th question.

**Figure 9:**
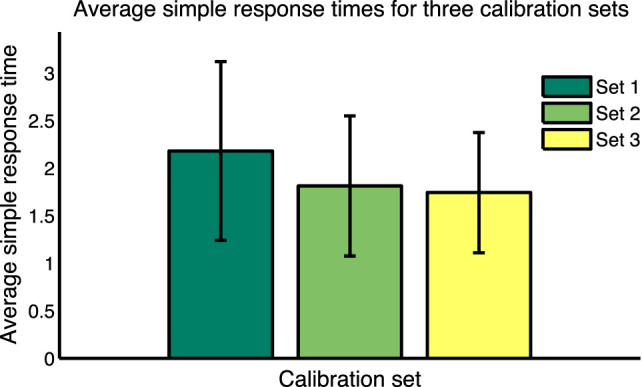
Average simple response times for the three calibration sets.

Average values for *T_s_* for all subjects for the 15 calibration questions in each of the three calibration sets are shown in Figure 9. Balanced one-way ANOVA for the three calibration sets showed that *T_s_* for Set 1 is considerably longer than for Set 3 (*p* < 0.05), indicating a learning effect through the trial. To take this learning effect into account, $\left(T_{s}^{i}\right)$ for each response for each subject was calculated by interpolating between average response times for the calibration sets for each subject. This calculation—for the first 72 test cases—is shown in Equation 2. The same approach was used for the second 72 test cases but using response times for calibration Sets 2 and 3.

(2) $$T_{s}^{i}={\bar{T}}_{s}(Set1)+\frac{{\bar{T}}_{s}(Set2)-{\bar{T}}_{s}(Set1)}{72}*i$$

where $T_{s}^{i}$ = simple response time for the *i*th question. ${\bar{T}}_{d}$ for the three questions are shown in Table 11.

**Table 11:** Average response time in seconds, showing the difference between when question is presented first or second/third.

	“Group”	“Order”	“Swarm”
Overall	0.83	1.76	0.97

**Order of question**			
Asked first	1.39	2.69	1.70
Asked second or third	0.55	1.29	0.61
*δ* t	0.85	2.15	1.39

**Sex of subject**			
Female	0.72	1.84	0.86
Male	0.91	1.70	1.06

**Rule-sets**			
Attract	1.03	2.72	1.29
Attract + Repel	0.83	2.10	1.22
Attract + Align + Repel	0.64	1.60	0.83
Align	1.24	1.66	0.63
Repel	0.62	1.27	0.92
Attract + Align	1.06	1.48	1.13
Repel + Align	1.04	1.90	0.88
No rules	0.71	1.07	0.79

**V_max_**			
1.0	0.77	1.96	1.06
2.0	0.83	1.66	0.96
4.0	0.88	1.65	0.90

#### 3.3.1 Comparison of “Swarm”, “Group,” and “Order” Decision Times

Balanced one-way ANOVA of average *T_d_* times for all test-sets for all subjects show that *T_d_* for the “order” question is significantly longer than for the “group” and “swarm” questions (*p* < 0.05). A possible explanation for the longer decision time for the order question is that the “order” decision is based on assessment of the relative velocities of particles which requires an integration of position information over time. The group decision, in contrast, does not require an integration over time, a decision can be made based on a snapshot in time. If the “swarm” decision is based on a combination of a “group” assessment and an “order” assessment, it would follow that the “swarm” decision would also take longer than the “group” decision. Results in Table 11 show that this is not the case—*T_d_* for the “swarm” decision is not significantly longer than for the “group” decision. An alternate explanation is necessary.

Observation of subjects during the trial showed that many subjects tended to lean forward and pay closer attention to the display when they were responding to the “order” question. This was not observed for the “swarm” or “group” questions. The “group” and “swarm” decisions may therefore be based on a broad, “holistic” assessment of behavior rather than paying close attention to individual particles which may be the case for the “order” decision. There may be similarities between the processes involved in the identification of swarming and the identification of biological motion. Johansson (1973) showed that subjects could identify biological motion—in this case humans walking and running—using 8–10 small number of dots attached to human limbs. Casile and Giese (2005) observed that such identification of biological motion could be performed by exploiting relatively simple neural circuits that identify optic flow patterns. If the swarming decision is based on a holistic assessment of behavior it may perhaps use the same simple neural circuits, leading to a fast decision time.

#### 3.3.2 Effect on Decision Times of Order in Which Questions Were Asked

*T_d_* was also analyzed to determine whether it was affected by the order in which questions were asked. Table 11 shows the *T_d_* results for the “group”, “order,” and “swarm” decisions depending on whether the question was asked first or second/third in the trial. Table 11 also shows the average difference (*δ*t) between asking the question first and later. One-way balanced ANOVA shows that *T_d_* for the “group”, “order,” and “swarm” decision are significantly longer when the question is asked first (*p* < 0.05) compared with second or third. This result suggests that subjects require a period of “familiarization”—around 1 to 2 s—before they make their first decision.

#### 3.3.3 Effect of Rule-Set on Decision Times

**Figure 10:**
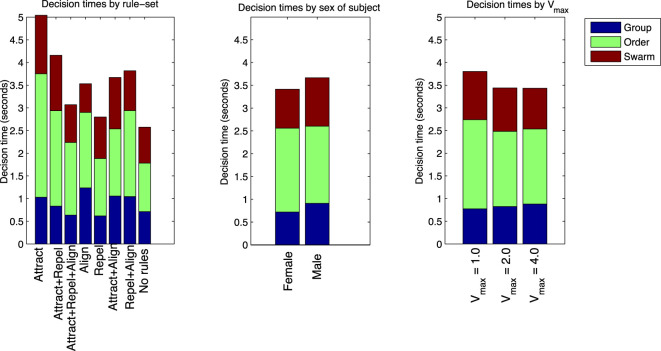
*T_d_* for “group”, “order,” and “swarm” questions, categorized by rule-set, sex of subject, and *V_max_*.

Table 11 and Figure 10 also show the *T_d_* results classified by the rule-sets of each of the test cases. Balanced one-way ANOVA for the decision times by rule-set showed no difference for the “group” or “swarm” questions. For the “order” question, *T_d_* was considerably longer for the Attract rule-set compared with the Attract + Align + Repel, Repel, and No rules rule-sets (*p* < 0.05). This result suggests that the time required to make an “order” decision is dependent on the nature of the dynamics in the model. This is not a simple differentiation between swarming and non-swarming behavior, however, as the three rule-sets with the shorter “order” decision times comprise both swarming and non-swarming cases.

#### 3.3.4 Effect of Sex of Subject on Decision Times

Table 11 and Figure 10 show the *T_d_* results classified by the sex of the subjects. Balanced one-way ANOVA of *T_d_* showed no significant difference between males and females for the “order” question, but *T_d_* was significantly shorter for females for both the “group” and “swarm” questions (*p* < 0.05). As for the identification of swarming, this result suggests that there is a significant gender effect in performance on the swarming identification task in this study.

#### 3.3.5 Effect of *V_max_* on Decision Times

Table 11 and Figure 10 show the *T_d_* results classified by *V_max_* for each of the test cases. Balanced one-way ANOVA for the decision times by *V_max_* showed no difference for any of the three questions based on *V_max_*.

The current boids model—and mathematical models of behavior developed later in this paper—provides a useful basis to further investigate differences in time taken to make decisions in relation to swarming motion. Further testing could include observation of subject behavior during testing. fMRIT analyses could also be conducted to determine whether different decision strategies and different brain regions are involved in making the three different decisions.

## 4 Conclusion and Future Work

In this paper, we showed that human subjects identify swarming behavior for a wide range of control parameters of a simplified version of the boids model. The majority of subjects identify swarming behavior where swarming is predicted by previous studies using objective measures. There is no uniform swarming/not-swarming decision across all subjects. Three factors that affect subjects’ identification of swarming behavior that we have identified are as follows: the presence or absence of the Attract, Align, and Repel forces; the maximum velocity of the particles; and the sex of the subject. The results indicate there are degrees of “swarminess” for cases produced by the model and individual subjects have different “swarminess” thresholds for identifying swarming behavior. The study shows that females have a lower “swarminess” threshold than males. This finding is consistent with previous visual perception studies that have found a higher level of performance by female subjects. It has been suggested, however, that rather than directly an effect of sex of subject, the difference is the result of brain lateralization effects with males tending to be more lateralized than females.

Analysis of response times for “swarming”, “grouped,” and “ordered” questions and informal observations of subject behavior during the subjective study suggest that different strategies, and potentially different areas of the brain, are being used to make the three decisions. The results suggest that the “swarming” and “grouped” decisions are based on a holistic assessment of behavior. The “ordered” decision may, in contrast, be based on a detailed assessment of behavior of individual particles.

The relatively simple swarming model of Reynolds used in this study together with the quantitative measures that characterize the behavior produced by the model are useful examples of how applying relatively simple mathematical models to the social sciences can help better understand individual human behavior. The simple models can provide a mathematically unbiased approach for better understanding the neurocognitive basis of dynamic visual pattern perception and potentially help explain individual perception differences, including those based on sex and brain lateralization.

## Ethics Statement

This study was carried out in accordance with the recommendations of UNSW Canberra Human Research Ethics Advisory Panel with written informed consent from all subjects. All subjects gave written informed consent in accordance with the Declaration of Helsinki. The protocol was approved by the UNSW Canberra Human Research Ethics Advisory Panel HREAP A-14-32.

## Author Contributions

JH conducted the study. KM and HA assisted with the design and revised the paper.

## Conflict of Interest Statement

The authors declare that the research was conducted in the absence of any commercial or financial relationships that could be construed as a potential conflict of interest.
